# Assessment of NADH/NAD^+^ Redox Imbalance in Psoriatic Lesions Using the FMSF Technique: Therapeutic Aspects

**DOI:** 10.3390/s23218718

**Published:** 2023-10-25

**Authors:** Jerzy Gebicki, Tomasz Filipiak, Andrzej Marcinek, Anna Wozniacka

**Affiliations:** 1 Institute of Applied Radiation Chemistry, Lodz University of Technology, 90-924 Lodz, Poland; andrzej.marcinek@p.lodz.pl; 2 Angionica Ltd., 90-924 Lodz, Poland; tomasz.filipiak@angionica.com.pl; 3 Department of Dermatology and Venereology, Medical University of Lodz, 90-419 Lodz, Poland; anna.wozniacka@umed.lodz.pl

**Keywords:** psoriasis, NADH/NAD^+^ redox imbalance, NADH fluorescence, FMSF technique

## Abstract

Mitochondrial dysfunction has been linked to psoriasis, and it may be an important underlying factor contributing to this disease. However, a precise methodology for assessing mitochondrial dysfunction has yet to be developed. One promising approach is to measure NADH autofluorescence from the affected skin areas. In this study, we show that Flow-Mediated Skin Fluorescence (FMSF) can be used for the non-invasive assessment of mitochondrial dysfunction in psoriasis. The fluorescence level at baseline and the half-time of ischemic growth (t_1/2_) derived from the FMSF traces can be used for the non-invasive assessment of NADH/NAD^+^ redox imbalance in psoriatic lesions compared to unaffected skin. These results are supported by an analysis of the key FMSF parameters: Reactive Hyperemia Response (RHR) and Hypoxia Sensitivity (HS). This method not only contributes to understanding the biochemical processes involved in the etiopathogenesis of psoriasis, but it also provides a basis for identifying new drug targets and improving the treatment process.

## 1. Introduction

Psoriasis is a chronic disease with a genetic predisposition that affects about 2% of the population. The etiopathogenesis of the disease is complex and not fully understood. Although multiple clinical types of psoriasis have been reported, most scientific research, including the present study, refers to the plaque variant, which affects up to 90% of all psoriatic patients. The hallmark of the disease is sustained inflammation that leads to uncontrolled keratinocyte proliferation. It is conjectured that disturbances in the innate and adaptive cutaneous immune responses are responsible for the development and maintenance of inflammation. 

Recent research indicates that mitochondrial dysfunction may also be an important factor in the development of the disease [1,2]. The mitochondria are involved in essential cell functions such as regulation of calcium homeostasis, innate immune and inflammatory responses, and apoptosis. 

A reliable diagnostic tool is therefore needed to assess mitochondrial dysfunction. One promising approach is to measure NADH autofluorescence [3]. Wollina et al. have shown that the intensity of NADH fluorescence is much lower in psoriatic lesions compared to unaffected skin [4]. This points to the presence of a NADH/NAD^+^ redox imbalance in psoriatic lesions, assuming that skin pigmentation is similar in lesioned and unaffected skin regions. However, it is well known that melanin concentrations in the skin seriously affect the measured fluorescence intensity [5,6]. The measured level of skin NADH fluorescence should therefore be used with caution for the characterization of mitochondrial dysfunction.

Flow-Mediated Skin Fluorescence (FMSF) is a new, non-invasive technique for assessing vascular circulation and metabolic regulation [7,8,9]. It measures the stimulation of the vascular circulation in response to post-occlusive reactive hyperemia based on dynamical changes in NADH fluorescence from skin tissue. Very recently, the FMSF technique has been used to analyze mitochondrial dysfunction in patients with newly diagnosed primary hypertension based on the ischemic phase of skin NADH dynamics [10]. This approach is particularly promising because the dynamics of NADH ischemic growth is independent of skin pigmentation.

In this report, we provide unambiguous evidence of NADH/NAD^+^ redox imbalance in psoriatic lesions based on an analysis of recorded FMSF traces. This observation, which was independent of skin pigmentation, clearly indicates that the NADH–NAD^+^ equilibrium is shifted toward oxidation in psoriatic lesions. The therapeutic implications of this observation are discussed.

## 2. Materials and Methods

### 2.1. Study Population and Clinical Characteristics

The studied population consisted of eight psoriatic patients newly admitted to the university hospital. The FMSF measurements were performed on the first day, before any topical treatment had been introduced that might interfere with the fluorescence measurements. Two patients, from whom FMSF traces were successfully collected from forearm skin with psoriatic lesions and from a closely located, unaffected area, were selected for detailed analysis. In these two patients, the measured psoriatic lesions fully covered the optical window surface. One of the patients (code 0004L) was a 50-year-old male. The other patient (code 0005L) was a 24-year-old female. Both patients had been suffering from psoriasis for 1–3 years. The diagnosis was confirmed by histopathological examination.

The control group consists of 16 healthy volunteers (7 females and 9 males; average age: 40.2 +/− 10.1 years).

### 2.2. Brief Description of the FMSF Technique and the Measurement Protocol

Measurements were performed using AngioExpert, a device constructed by Angionica Ltd. The AngioExpert device uses the Flow-Mediated Skin Fluorescence (FMSF) technique, which measures changes in the intensity of nicotinamide adenine dinucleotide (NADH) fluorescence from the skin on the forearm as a response to blocking and releasing blood flow. The skin is the largest organ of the human body, and it is characterized by a specific metabolism. The epidermal layer of skin is not directly vascularized, and oxygen and nutrients are transported from the dermis by diffusion. Therefore, epidermal cell metabolism can be considered a unique and sensitive marker of early dysfunction in vascular circulation and metabolic regulation.

The measurement protocol has been described elsewhere [9]. For the analysis of the results, four parameters were used. Two fluorescence parameters, which measure the fluorescence level at baseline and the half-time of ischemic growth (t_1/2_), characterize the FMSF trace. The other two parameters, which measure the Reactive Hyperemia Response (RHR) and Hypoxia Sensitivity (HS) expressed as log(HS), have been demonstrated and discussed previously [8,9]. The RHR is a unique parameter based on a combination of both the ischemic and hyperemic parts of the measured FMSF trace. It characterizes the endothelial function related predominantly to the production of nitric oxide (NO) in the vasculature due to reactive hyperemia. The log(HS) parameter characterizes the intensity of myogenic microcirculatory oscillations (0.052–0.15 Hz) observed on the reperfusion line. It represents the response of microcirculation to transient ischemia.

## 3. Results and Discussion

**Figure 1:**
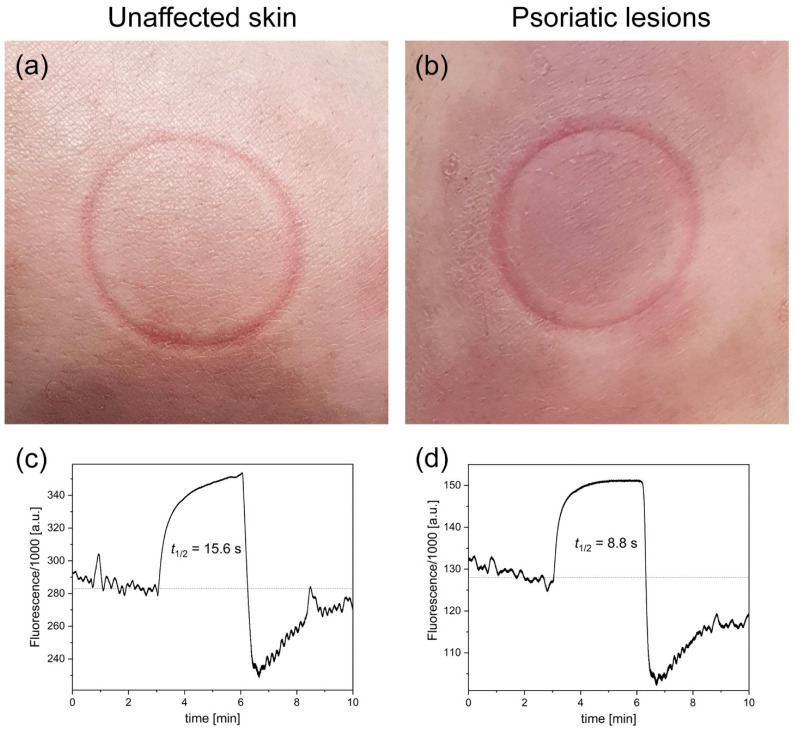
Photographs of unaffected skin (**a**) and psoriatic lesions (**b**) for a single patient (code 004L) and FMSF traces measured from these areas (**c**,**d**).

Figure 1 presents example FMSF traces collected for psoriatic lesions and unaffected skin from the patient (code 004L), along with photographs of the studied skin areas, including characteristic imprints from the optical window of the AngioExpert diagnostic device. The fluorescence level at baseline value collected from unaffected skin was 284,000 a.u., and the half-time (t_1/2_) of ischemic growth was 15.6 s. The results for the psoriatic lesions display two characteristic features compared to the unaffected skin: a lower fluorescence level at baseline (128,000 a.u.) and a shorter half-time (t_1/2_) of ischemic growth (8.8 s). Very similar results were obtained for the other patient (code 005L). The results for both patients are presented in Table 1, along with the measured FMSF parameters. As can be seen, the FMSF parameters collected from psoriatic skin were lower than those collected from unaffected skin. A particularly pronounced difference is visible in the log(HS) parameter.

The lower fluorescence level at baseline values recorded for psoriatic lesions compared to unaffected skin supports previous observations by Wollina et al. [4]. The important new finding made in the present study is based on the shorter half-time of ischemic growth recorded for psoriatic lesions. This observation, which was independent of skin pigmentation, clearly indicates that the NADH–NAD^+^ equilibrium is shifted toward oxidation in psoriatic lesions and that transient ischemia caused by blocking of the blood flow in the brachial artery results in a very rapid reduction of NAD^+^ to NADH in skin keratinocytes. A shorter half-time of ischemic growth (t_1/2_) in psoriatic lesions was seen in all studied patients, and in an extreme case (patient code 008L), it was as low as 4.2 s. The half-time of ischemic growth calculated for the healthy group (n = 16) is 15.8 ± 2.9 s. It can generally be assumed that a t_1/2_ of ischemic growth in the recorded FMSF traces below 10 s is indicative of mitochondrial dysfunction, represented by a shift of NADH/NAD^+^ redox imbalance toward oxidation. The lower log(HS) parameters collected from psoriatic lesions compared to unaffected skin seem to confirm this assumption, as the highly oxidated skin keratinocytes seen in psoriatic lesions should have a lower response to transient hypoxia. Thus, the observed fluorescence and FMSF parameters presented in Table 1 can be used for the non-invasive assessment of mitochondrial dysfunction.

A particularly interesting observation concerns a lower level of fluorescence at baseline collected from psoriatic lesions compared to a closely located unaffected skin area for each of the eight psoriatic patients studied. The difference in the fluorescence levels between these two areas related to the fluorescence level collected from unaffected skin varied from 13.4% to 54.3%, with an average value of 36.6%. This observation suggests a distinguishable NADH/NAD^+^ redox imbalance in psoriatic lesions characteristic of each studied patient. An important hypothesis can be formulated about whether such a redox imbalance can be associated with a disease status. As the measurements of NADH fluorescence from the skin are simple, fast, and fully non-invasive, it is worth clinically verifying such a hypothesis. It may turn out that such an approach can be useful in diagnostics and the monitoring of psoriatic patients. For that purpose, a mobile optical detector should be constructed as a use of the AngioExpert diagnostic device limits observation of psoriatic lesions only to the forearm area.

The evidence that psoriasis lesions show an NADH/NAD^+^ redox imbalance toward oxidation may have important therapeutic implications. A deviation represented as a shift in the fluorescence and FMSF parameters collected from unaffected skin vs. psoriatic lesions could potentially be used as a measure of disease status and progress. This could, in turn, allow for a better understanding of therapeutic options to treat psoriasis.

The identification of biomarkers for psoriasis based on metabolomics suggests the operation of the compensatory effect, involving activation of glycolytic activity resulting in elevated levels of lactic acid [11,12]. Indeed, the NADH/NAD^+^ ratio may correlate with the pyruvate/lactate ratio. Surprisingly, potentiation of the NADH/NAD^+^ redox imbalance by increasing the level of NAD^+^ in psoriatic lesions could be considered a therapeutic option. We have already shown that the topical composition of NAD^+^ can effectively be used for the treatment of psoriasis, with effects comparable to conventional therapy with anthralin [13]. More recent research has confirmed that the augmentation of NAD^+^ can result in the amelioration of psoriasis-like dermatitis [14]. This effect is linked to elevated Sirt1 activity, which is an NAD^+^-dependent process. The therapeutic effect of hyperbaric oxygen in psoriasis can also be explained by the activation of the NADH/NAD^+^ redox imbalance [15]. On the other hand, such effects may also be explained as mitohormesis (a biological response where the induction of a reduced amount of mitochondrial stress leads to an increase in health and viability within a cell, tissue, or organism) [16].

The application of the presented methodology for the diagnosis of psoriasis is greatly facilitated by the fact that results can be collected from both psoriatic lesions and the unaffected skin of individual patients. However, research should be extended to evaluate the application of this methodology to other diseases and disorders.

## Figures and Tables

**Table 1:** Fluorescence and FMSF parameters measured for two psoriatic patients.

Patient’s Code	Fluorescence Parameters		FMSF Parameters	
	Fluorescence at Baseline (a.u.)	t_1/2_ (s)	RHR (%)	log (HS)
004L unaffected skin	284,000	15.6	43.9	1.59
004L psoriatic lesions	128,000	8.8	38.5	1.08
005L unaffected skin	412,000	12.9	37.5	2.08
005L psoriatic lesions	261,000	8.4	36.4	1.64

## Data Availability

Data presented in this paper are available on request.
